# Fusion Protein Vaccine Based on Ag85B and STEAP1 Induces a Protective Immune Response against Prostate Cancer

**DOI:** 10.3390/vaccines9070786

**Published:** 2021-07-13

**Authors:** Linpei Guo, Hui Xie, Zheng Zhang, Zhun Wang, Shuanghe Peng, Yuanjie Niu, Zhiqun Shang

**Affiliations:** 1 Tianjin Institute of Urology, The Second Hospital of Tianjin Medical University, Tianjin 300211, China; guolinpei@tmu.edu.cn (L.G.); xhinfinit@tmu.edu.cn (H.X.); zhangzheng0602@126.com (Z.Z.); 18853734760@163.com (Z.W.); 2 Department of Pathology, The Second Hospital of Tianjin Medical University, Tianjin 300211, China; womiu001@163.com

**Keywords:** Ag85B, *Mycobacterium* *tuberculosis*, STEAP1, six-transmembrane epithelial antigen of the prostate 1, immune response, prostate cancer, castration-resistant prostate cancer (CRPC)

## Abstract

(1) Background: There are currently limited treatments for castration-resistant prostate cancer. Immunotherapy involving Sipuleucel-T has increasingly drawn attention for prostate cancer management. BCG plays a vital role in treating bladder cancer, mainly by inducing immune activation, but is rarely used for prostate cancer. (2) Methods: The TCGA database, PCR, and Western blotting were used to analyze the expression of STEAP1 in mouse and human tissues. Then, we constructed a fusion protein vaccine with *Mycobacterium tuberculosis* Ag85B and three repeated octapeptide epitopes of a six-transmembrane epithelial antigen of the prostate 1 (STEAP1_186-193_), Ag85B-3×STEAP1_186-193_. The uptake of the fusion protein vaccine by DCs was evaluated by confocal microscopy, and DC markers were detected using flow cytometry after incubation with the fusion protein. The immune response against prostate cancer was evaluated by the LDH assay and xenografts in vitro and in vivo. Then, the tumor microenvironment was determined using IHC and ELISA. In addition, the epitope was mutated using CRISPR-Cas9 to illustrate that the fusion protein elicited immunization against STEAP1. (3) Results: The TCGA database analysis, PCR, and Western blotting showed that STEAP1 was highly expressed in human and murine prostate cancer. After the uptake of the purified fusion protein vaccine by DCs, CD11c, CD80, CD86, and MHC II were upregulated and triggered a cytotoxic T lymphocyte (CTL) response against TRAMP-C1 and RM1 cells in vitro. Furthermore, the fusion protein vaccine inhibited tumor growth and improved the tumor microenvironment in vivo, with more CD3^+^ cells and fewer FOXP3^+^ cells in the tumor. Serum IFN-γ and IL-2 were significantly higher than in the control group, while IL-4 expression was lower, indicating that the fusion protein vaccine activated Th1 immunity. The immune response against prostate cancer was greatly suppressed when the antigen targets were knocked out using CRISPR-Cas9. (4) Conclusion: In summary, our results provide the first evidence that a vaccine based on a fusion protein consisting of Ag85B and a prostate cancer octapeptide epitope with complete Freund’s adjuvant (CFA), triggers a robust immune response and inhibits tumor growth in murine prostate cancer.

## 1. Introduction

For several years, prostate cancer has represented the greatest proportion of new tumor cases and has been the leading cause of cancer deaths in western men [1,2]. Prostatectomy and hormone deprivation therapies are effective in early-stage prostate cancer patients, but the existing treatments and efficacy are not promising once a patient has advanced to castration-resistant prostate cancer (CRPC), which has a poor prognosis. Historically, based on the degree of metastatic disease and the occurrence of symptoms, the average survival of CRPC patients has been estimated to be 9–36 months [3,4]. Therefore, new treatments for prostate cancer are needed. In recent decades, the rapid development of tumor immunology has inspired prostate cancer treatment.

Sipuleucel-T (*Provenge*) was approved by the FDA in 2010, made from GM-CSF (granulocyte-macrophage colony-stimulating factor) and the prostate tumor-associated antigen (TAA) PAP (prostatic acid phosphatase). It activates an antitumor response against PAP [1,5]. As a tumor-associated antigen, PAP is significantly upregulated in prostate cancer, while it has a low expression in normal tissues, including the prostate. In this study, six transmembrane epithelial antigens of prostate 1 (STEAP1) had a similar expression pattern and it was selected as a TAA for the prostate. STEAP1 has low expression in normal prostate but is overexpressed in a variety of carcinomas, such as bladder cancer, breast cancer, melanoma, and Ewing’s sarcoma cancer [6,7,8]. The unique expression pattern of STEAP1 makes it an excellent target for tumor vaccines. Krupa et al. immunized C57BL/6 and transgenic adenocarcinoma mouse prostate (TRAMP) mice against STEAP1 using a modified vaccinia ankara (MVA) and, later, STEAP1-MVA. The results showed that the vaccine reduced the tumor size by 76.5% compared to in the control group, and increased T-cell infiltration in prostate cancer [9]. A DNA vaccine strategy based on STEAP1 was evaluated as a possible treatment for prostate cancer.

Ag85B, a 325 aa protein, is highly expressed in all mycobacteria and is one of the main protein components that induce a human immune response against mycobacteria. Ag85B is involved in the synthesis of mycolic acids in the cell wall and induces a strong Th1-type immune response in hosts [10,11]. Previous studies have shown that an Ag85B fusion protein, such as Ag85B-MPT64190–198-Mtb8.4, is able to boost immunity against *Mycobacterium tuberculosis*. However, the effects of the Ag85B and TAA fusion protein on the tumor-specific CTL response are still unclear.

In this study, a fusion protein vaccine based on Ag85B and STEAP1_186-193_ was expressed and purified in E. coli, then the CTL response and tumor growth inhibition for prostate cancer were evaluated.

## 2. Materials and Methods

### 2.1. Antigen Expression Analysis with Polymerase Chain Reaction (PCR)

First, 12- and 24-week-old TRAMP mice (The Jackson Laboratory, Bar Harbor, ME, USA) were sacrificed, and the organs were minced and immediately immersed in TRIzol reagent (Thermo, Waltham, MA, USA). A glass grinder was used to grind the tissues into homogenate in the TRIzol for RNA extraction. Reverse transcription was performed, and the mRNA levels were analyzed using polymerase chain reaction (PCR) with specific primers (Table 1). The PCR was performed with 30 cycles of replication (95 °C, 30 s; 57 °C, 30 s; 72 °C, 30 s) after 5 min of denaturation at 95 °C, followed by a final extension at 72 °C for 3 min. A 10 μL volume of the reaction product was separated in a 1.5% agarose gel.

### 2.2. Western Blotting

Protein samples were separated by SDS-PAGE and then electroblotted onto a PVDF membrane, which was incubated with a primary antibody (Anti-STEAP1, Invitrogen, Waltham, MA, USA, 1:1000; Anti-6×His, Abcam, Cambridge, UK, 1:1000; Anti-GAPDH, Abcam) at 4 °C overnight after blocking it in 5% milk at room temperature for 30 min. After washed with TBST buffer, the membrane was then incubated with an HRP-conjugated secondary antibody (BBI, Shanghai, China) for 1 h at room temperature following a TBST wash for 30 min. The protein bands were then visualized using ECL reagent (Sigma-Aldrich, Burlington, CA, USA).

### 2.3. Expression Plasmid Construct

DNA fragments of Ag85B and a linker were obtained from *Mycobacterium tuberculosis* by PCR. A flexible linker sequence was added to the amino terminus of the protein (the primer sequences are listed in Table 1, F2 + R2). GENEWIZ Co., Ltd (Suzhou, China). synthesized the antigen peptides, 3×STEAP1_186-193_. The sequence of 3×STEAP1_186-193_ is as follows: CGTTCCTACCGTTACAAACTGCTGCGTTCCTACCGTTACAAACTGCTGCGTTCCTACCGTTACAAACTGCTGTAG. Overlap PCR was used to link the two fragments of Ag85B + link and 3×STEAP1_186-193_ into a whole fragment (the primer sequences are listed in Table 1, F2 + R3). The whole fragment was inserted into pET28a between the NheI and XhoI sites using an In-Fusion cloning kit (638910, Takara, San Jose, CA, USA), forming the expression plasmid pET28a-Ag85B-3×STEAP1_186-193_. All the recombinant plasmids were verified using Sanger sequencing.

### 2.4. Fusion Protein Preparation

pET28a-Ag85B-3×STEAP1_186-193_ was transformed into Rosetta(DE3) cells, which were incubated in LB medium with kanamycin (100 μg/mL) while shaking (200 rpm/min) at 37 °C overnight. Then, 2.5 mL of culture was mixed with 250 mL of LB medium containing 100 μg/mL kanamycin and transferred in a 1000 mL flask and incubated at 30 °C until the OD_600_ reached 0.7. Isopropyl β-D-1-thiogalactopyranoside (IPTG, Sigma-Aldrich, St. Louis, MO, USA) was added to the medium to a final concentration of 1 mM to induce protein expression. After six hours, the Rosetta (DE3) cells were centrifuged at 12,000× *g* at 4 °C for 10 min to collect the precipitate. Then, the precipitate was washed twice using PBS and dissolved in a 6 M guanidine hydrochloride solution. A Ni-NTA Purification System was used to purify the fusion protein. The protein dissolved in guanidine hydrochloride was put in a 25 KD dialysis membrane (BBI, Shanghai, China), and the concentration of guanidine hydrochloride outside the dialysis membrane was gradually reduced to induce protein refolding.

### 2.5. Fusion Protein Analysis with Coomassie Brilliant Blue

A bacterial suspension (1 mL) was centrifuged at 12,000× *g* for 10 min and resuspended in 100 μL of PBS. Then, 25 μL of 5×loading buffer was added and the mixture was put into a metal bath at 95 °C for 10 min. Next, 10 μL of the mixture was loaded per well in a 10% SDS-PAGE gel. The protein bands were visualized using Coomassie Brilliant Blue R-250 (BBI, Shanghai, China).

### 2.6. Cells

The murine cell line DC2.4 (ATCC, USA, DCs, H-2^b^) was incubated in RPMI-1640 medium with 1% Penicillin-Streptomycin (Solarbio, Beijing, China), 0.055 mM β-mercaptoethanol (Solarbio, Beijing, China), and 10% fetal bovine serum (Biological Industries, Beit haemek, Israel). The murine prostate cancer cell line (TRAMP-C1(ATCC) and RM1 (ATCC), H-2^b^, obtained from C57BL/6) was incubated in RPMI-1640 medium with 1% Penicillin-Streptomycin, and 10% fetal bovine serum. All the experiments were performed using mycoplasma-free cells.

### 2.7. Fusion Protein Labeled with 5-Aminofluorescein (5-AF)

First, 10 mg of fusion protein, 3 mg of EDC (E106172, Aladdin, Shanghai, China), and 3 mg of NHS (H109330, Aladdin) were added to 2 mL of MES buffer (pH = 5.6) and left to react for 15 min at room temperature. Then, 1 mg of 5-aminofluorescein (A110141, Aladdin) was added into the reaction. After 8 hours, the fusion protein labeled with 5-aminofluorescein was separated by ultrafiltration (C7719-3kDa, Millipore) at 4000× *g* for 20 min.

### 2.8. Uptake of Fusion Protein by DCs

DCs were seeded into confocal dishes with 1 mL of complete medium and 10 μg/mL fusion protein labeled with 5-AF. For the control, DCs were incubated with complete medium supplemented with 1 μg/mL 5-AF. After 12 h of incubation, the cells were fixed with 4% paraformaldehyde at 4 °C for 30 min and then stained with DAPI (C0065, Solarbio, Beijing, China). Confocal microscopy (FV1000, Olympus, Tokyo, Japan) was used for observation.

### 2.9. Flow Cytometry

DCs were seeded into 6-well plates with 3 mL of complete medium, and 10 μg/mL purified fusion protein. After 24 h of incubation, we harvested 1 × 10^6^ target cells in 100 µL of cell staining buffer and blocked Fc-receptors with 0.25 μg of TruStain FcX™ PLUS (Biolegend, San Diego, CA, USA) antibody on ice for 10 min. Conjugated fluorescent antibodies were then added into cell staining buffer at optimum concentrations and incubated at 4 °C for 30 min: Percp-cy5.5-CD11c (117327, Biolegend), PE-CD80 (104707, Biolegend), PE-cy7-CD86 (105013, Biolegend), and APC-MHC II (116417, Biolegend).

### 2.10. Cytotoxicity Assay

For the preparation of effector T cells, C57BL/6 mice were immunized with 50 μg fusion protein vaccines, followed by boosters 7 days later, four times. Splenocytes were harvested 7 days later and incubated with different concentrations of fusion protein overnight.

A Cytotoxicity Detection Kit (LDH, Roche) was used according to the instructions to determine the effector T cells’ cytotoxicity against tumor cells. The ratio of T cells to tumor cells was 20:1. After incubation for 8 h, the absorbance at 490 nm was measured. Cytotoxicity (%) = ((effector − target cell mix − effector cell control) − low control)/(high control − low control) × 100%.

### 2.11. Mice

C57BL/6 (H-2^b^, HFKbio, Beijing, China) mice were used in all the experiments. We established RM1 and TRAMP-C1 xenograft tumor models by implanting 5 × 10^6^ tumor cells into the groin of a C57BL/6 mouse. After 2 days, the treatment groups were immunized by the intradermal injection of 50 μg of fusion protein and 100 μL of Freund’s complete adjuvant (Sigma, St. Louis, MO, USA) in the limb areas near the lymph nodes (dissolved in 200 μL of PBS), while the control groups were immunized using PBS and 100 μL of Freund’s complete adjuvant. The immunization boost for the RM1 xenograft tumors was performed on the 9th and 16th days after the first immunization, and the mice were sacrificed on the 23rd day, while that for the TRAMP-C1 xenograft tumors was performed on the 9th, 16th, and 23rd days after the first immunization, and the mice were sacrificed on the 30th day because the tumor growth in vivo was slower than that for the RM1 cells. The tumor size was measured every 3 days using Vernier calipers 7 days after transplantation. The tumor volume was calculated using V = L × W^2^ × 0.5 (V = volume, L = length, and W = width). All the animal assays were approved by the Animal Ethical and Welfare Committee of Tianjin Medical University.

### 2.12. Immunohistochemistry (IHC)

The IHC assays were performed using an IHC kit (PV6000, ZSBIO, Beijing, China), and all the steps were performed according to the instructions. Importantly, the antibody (Anti-FOXP3, Abcam, ab215206, 1:500; Anti-CD3, Abcam, ab135372, 1:500) was incubated overnight. After staining, the IHC results were determined using ImageJ (v1.8.0_172) in Windows.

### 2.13. Separation of Lymphocytes from the Spleen

The spleen was minced into small pieces and gently forced through a 100 μm-gauge stainless-steel mesh with a sterile syringe plunger to separate the splenocytes. The cell suspension was filtered using a 40 μm cell strainer and resuspended in ammonium chloride–potassium lysis buffer to remove the red blood cells. The mixture was added into Lymphocyte Separation Medium (P8860, Solarbio) and centrifuged at 800× *g* for 30 min at room temperature to separate the lymphocytes from the spleen.

### 2.14. T Cell Proliferation Assay

T cells were obtained from the spleens of the immunized or control C57BL/6 mice and were labeled with carboxyfluorescein diacetate succinimidyl ester (CFSE, Sigma). Then, the T cells were incubated with 10 μg/mL fusion protein for 3 days. The T cell proliferation was detected using flow cytometry. For CCK-8 assay, T cells obtained from the spleen of immunized or control mice were incubated with 10 μg/mL fusion protein in 200 μL medium for 3 days. 20 μL CCK-8 was added and incubated for 2 h at 37 °C, and the absorbance at 450 nm was measured. 

### 2.15. ELISA

The mice were sacrificed, and blood was collected and centrifuged to separate the serum. Then, IFN-γ (EMC101g.48, neobioscience, Shenzhen, China), IL-2 (EMC002.48, neobioscience), and IL-4 (EMC003.96, neobioscience) were quantitated using an ELISA kit according to the instructions.

### 2.16. STEAP1 Knockout Using CRISPR-Cas9

The knockout of STEAP1 was conducted using the lentiCRISPR-v2-puro system (52961, Addgene) and sgRNA: acagtctctcatacccaatg. The packaging plasmid and lentiCRISPR-v2-STEAP1 were then transfected into 293FT cells (R70007, ThermoFisher, Waltham, MA, USA). The lentivirus was purified using PEG8000 and used to infect TRAMP-C1 cells. Positive cells were cultured with 2 μg/mL puromycin, and monoclonal cells were sorted and verified using Sanger sequencing.

### 2.17. Statistical Analysis

The cytotoxicity, xenograft growth, and ELISA results were analyzed using two-tailed, paired Student’s *t*-tests. The survival was analyzed using log-rank tests. *p* less than 0.05 is considered statistically significant.

## 3. Results

### 3.1. STEAP1 Is Highly Expressed in Prostate Cancer and Gradually Increases as the Tumor Progresses

**Figure 1:**
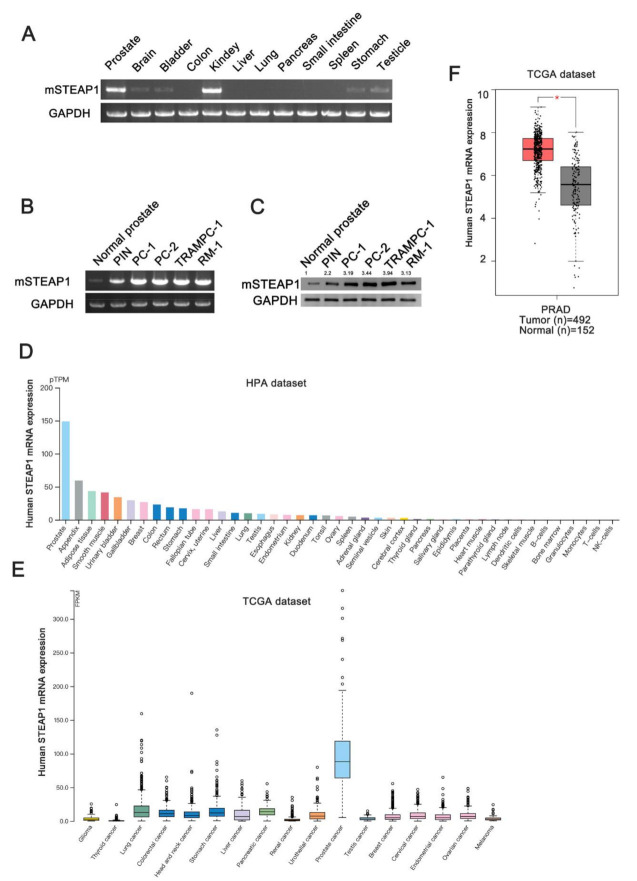
STEAP1 is highly expressed in the prostate cancer and gradually increases as the tumor progresses. (**A**) The mRNA expression of mouse STEAP1 in normal tissues. (**B**) The mRNA expression of mouse STEAP1 in normal prostate, prostatic intraepithelial neoplasia (PIN), prostate cancer (PC, TRAMP mouse), and prostate cancer cells of mouse. All PCR products were separated using 1.5% agarose gel. (**C**) Mouse STEAP1 protein expression in prostate and prostate cancer cells. (**D**) STEAP1 expression levels in normal human tissues and human cancers (**E**). (**F**) The expression of STEAP1 in human normal prostate and prostate cancer in TCGA database. * *p* < 0.05.

PCR assays were used to detect the STEAP1 mRNA expression level in each tissue of the mouse. The STEAP1 mRNA was highly expressed in the prostate and kidney but low in the brain, bladder, stomach, and testis, as shown in Figure 1A. Then, we evaluated the STEAP1 mRNA and protein expression in normal prostate tissues, prostatic intraepithelial neoplasia (PIN), prostate cancer (PC), and prostate cancer cell lines. PIN and PC were derived from the prostate of TRAMP mice at 12 and 24 weeks, respectively, and were confirmed by pathology. The results showed that STEAP1 expression gradually increased in the normal prostate, PIN and PC of the mouse (Figure 1B,C), which is consistent with previous research [12,13,14]. STEAP1 is a highly conserved protein, and the similarity between the human and mouse forms is as high as 80%. The Human Protein Atlas database (https://www.proteinatlas.org/ accessed on: 1 July 2021) showed that STEAP1 was highly expressed in normal human prostate tissues (Figure 1D, Figures S1, S2) and highly expressed in prostate cancer compared to other human tumors (Figure 1E). Then, we analyzed the expression of STEAP1 in the TCGA database, and the results showed that STEAP1 was overexpressed in human prostate cancer compared to adjacent normal prostate tissues (Figure 1F).

### 3.2. Preparation of Fusion Protein with pET28a

**Figure 2:**
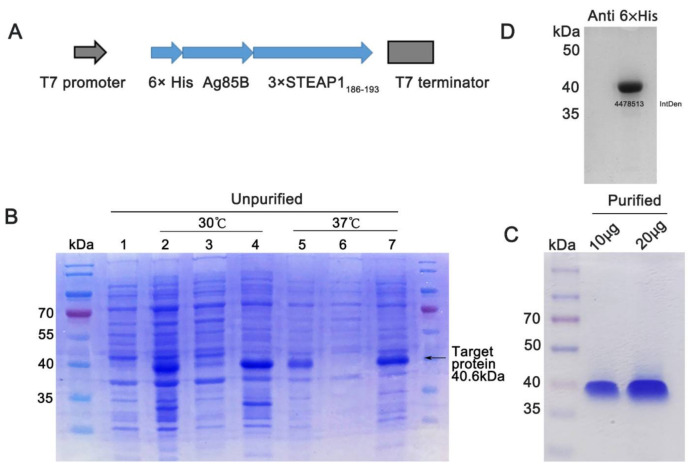
Preparation of fusion protein with pET28a. (**A**) Schematic of pET28a-Ag85B-3×STEAP1_186-193_ plasmid. Ag85B-3×STEAP1_186-193_ fragment was inserted into pET28a between NheI and XhoI sites using the In-Fusion cloning method. (**B**) 10% SDS-PAGE analysis of total protein of Rosetta(DE3); the protein was stained using Coomassie Brilliant Blue. Lane 1: Rosetta(DE3) control; Lane 2, total Rosetta(DE3) lysate, IPTG induced at 30 °C; Lane 3, Rosetta(DE3) lysate supernatant, IPTG induced at 30 °C; Lane 4, Rosetta(DE3) lysate precipitation, IPTG induced at 30 °C; Lane 5, total Rosetta(DE3) lysate, IPTG induced at 37 °C; Lane 6, Rosetta(DE3) lysate supernatant, IPTG induced at 37 °C; Lane 7, Rosetta(DE3) lysate precipitation, IPTG induced at 37 °C. (**C**) Coomassie Brilliant Blue staining of purified fusion protein. (**D**) Western blotting of purified fusion protein. IntDen: Integrated density.

The fusion protein expression plasmid pET28a-Ag85B-3×STEAP1_186-193_ was constructed and confirmed using Sanger sequencing (Figure 2A). The plasmid was then transferred into Rosetta (DE3) cells, used to express the fusion protein. Unfortunately, the fusion protein was expressed in the inclusion bodies (Figure 2B). Then, the fusion protein was purified and renatured according to the Ni-NTA agarose gel instructions and confirmed using Coomassie Blue Staining (Figure 2C) and Western blotting (Figure 2D).

### 3.3. Fusion Protein Vaccine Activated DC Cells and Elicited Efficient Cytotoxic T Lymphocyte (CTL) Responses In Vitro

**Figure 3:**
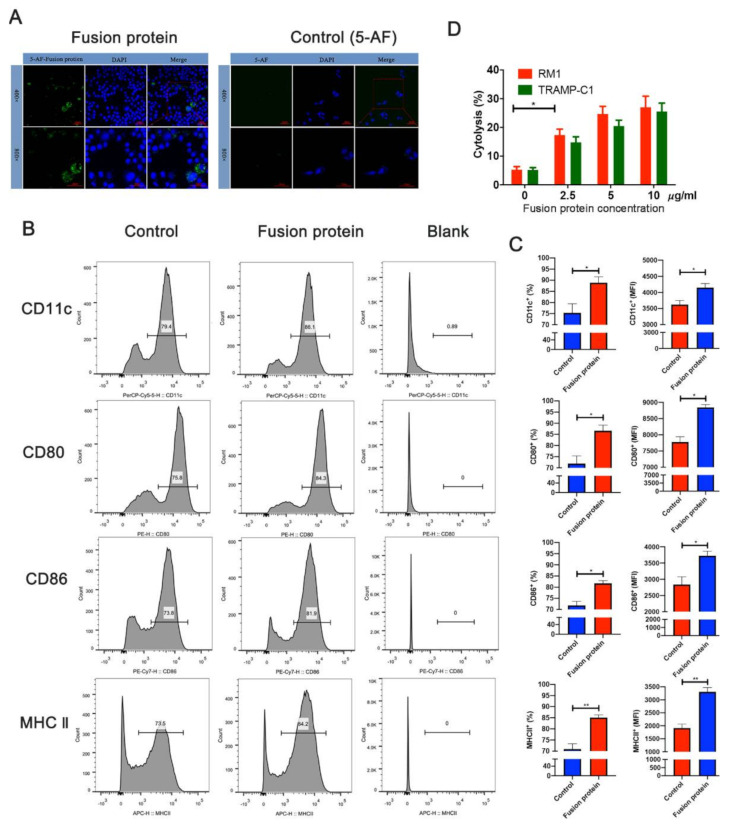
Fusion protein vaccine activates DCs and elicits efficient cytotoxic T lymphocyte (CTL) responses in vitro. (**A**) DCs taking up the fusion protein, which was labeled with 5-aminofluorescein (5-AF). (**B**) Flow cytometry analysis of protein markers in DCs. (**C**) Quantified results of the flow cytometric analysis. MFI, mean fluorescence intensity. (**D**) The lactate dehydrogenase-releasing cytotoxicity assay (LDH) was performed to determine the cytotoxic efficiency of effector T cells against RM1 and TRAMP-C1 cells at different concentrations of the fusion protein. * *p* < 0.05; ** *p* < 0.01.

Since antigen uptake is essential for the antigen presentation of DCs, we used confocal assays to verify that DC2.4 took up the fusion protein vaccine labeled with 5-aminofluorescein (Figure 3A). The myeloid DC marker CD11c and costimulatory factors CD86, CD80, and MHC II were assessed using flow cytometry after the fusion protein was incubated with the DCs for 24 h to determine whether the fusion protein activated DCs. The results showed that the fusion protein promoted the expression of CD11c, CD80, CD86, and MHC II, suggesting that the fusion protein activates DC cells (Figure 3B,C). The lactate dehydrogenase (LDH)-releasing cytotoxicity assay was performed to measure the cytotoxicity of effector T cells against prostate cells, and the results showed that the fusion protein could elicit an efficient cytotoxic T lymphocyte response against tumor cells overexpressing the STEAP1 protein (Figure 3D).

### 3.4. Immunization with Fusion Protein Inhibited Prostate Cancer Cells in Mice

**Figure 4:**
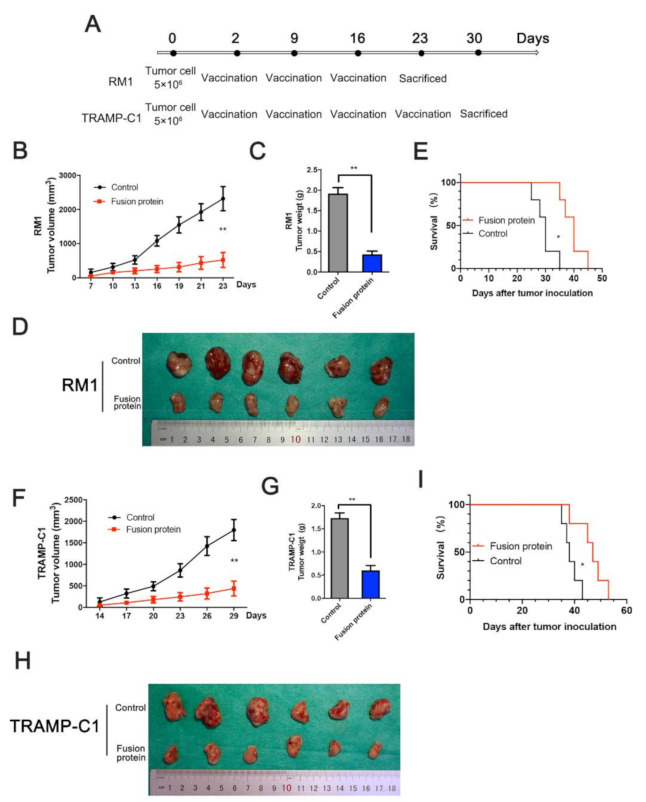
Immunization with fusion protein inhibited prostate cancer cells in mice. (**A**) Schematic diagram for the immunization and xenograft in MHC-matched C57BL6 mice. RM1 xenograft was immunized thrice, and TRAMP-C1 xenograft was immunized four times. (**B**) Tumor volume analysis for RM1 xenograft. (**C**,**D**) RM1 tumor weight measurement after the mice were sacrificed. (**E**) The Kaplan–Meier survival curves of RM1 model for different treatments. (**F**–**I**) Tumor growth analysis and TRAMP-C1 xenograft tumor in prophylactic settings. * *p* < 0.05; ** *p* < 0.01.

We established a xenograft tumor model by implanting 5 × 10^6^ tumor cells in a C57BL/6 mouse groin to confirm whether the fusion protein inhibited the tumor in vivo. The immunization boost for the RM1 xenograft tumors was performed after the first immunization on the 9th and 16th days, and the mice were sacrificed on the 23rd day (Figure 4A). After 23 days, the tumor growth curve and gravimetric analysis showed that the fusion protein significantly inhibited RM1 tumor growth and extended the survival time of tumor-bearing mice in vivo (Figure 4B–E). For the TRAMP-C1 xenograft tumors, the mice were sacrificed 7 days after four immunizations, and the tumors were weighed for analysis (Figure 4A). The inhibition of tumor growth by the fusion protein could be distinctly observed (Figure 4F–I). The results indicate that the fusion protein has a significant antitumor effect in mice.

### 3.5. Fusion Protein Mediated T Cell-Dependent Antitumor Effect

We examined the changes in immune cells in the tumor microenvironment to determine whether immunity played a role in tumor suppression. Tumor suppression experiments in mouse showed that TRAMP-C1′s inhibition efficiency was higher than that of RM1, and subsequent experiments were performed using TRAMP-C1 models.

**Figure 5:**
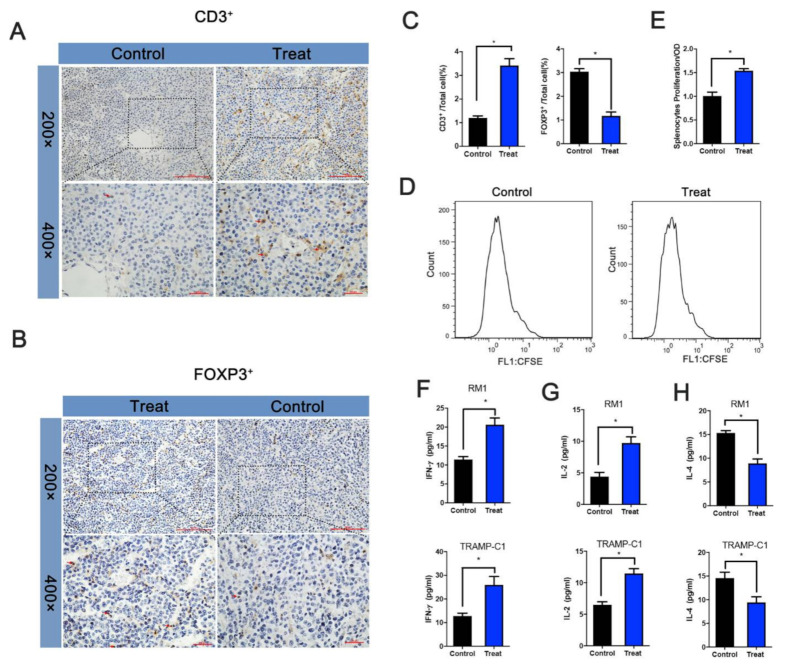
Fusion protein mediated T cell-dependent antitumor effect. (**A**) Distribution of CD3^+^ cells and FOXP3^+^ cells. (**B**) in TRAMP-C1 xenograft. (**C**) Statistical analysis of the proportion of positive cells among all cells. (**D**) Spleen cell proliferation assays. After the mice were sacrificed, spleen cells were isolated and labeled with CFSE and incubated with the fusion protein. Cell proliferation was then identified using flow cytometry or CCK8 (**E**). (**F**–**H**) Determination of IFN-γ, IL-2, and IL-4 in the RM1/TRAMP-C1 xenograft model serum after sacrificing the mice. * *p* < 0.05.

The IHC assays identified CD3^+^ and FOXP3^+^ cells in xenograft tumors, and the results showed that the proportion of CD3^+^ cells in the treatment group was higher, while that of FOXP3^+^ cells was lower (Figure 5A–C). Splenocytes were stained using 5(6)-carboxyfluorescein diacetate succinimidyl ester (CFSE) and incubated with the fusion protein for 3 days to verify specifically activated splenocytes from the treatment and control groups. The results showed that the treatment group’s splenocytes underwent multiple mitoses, while the splenocytes from the control group had no obvious mitosis (Figure 5D). In addition, the splenocyte CCK8 assay showed that the treatment group’s absorbance was significantly higher than the control group’s (Figure 5E).

Cell cytokines play an essential role in CTL. IL-2, IFN-γ, and IL-4 were further examined using ELISA to understand the changes in cell cytokines during immunization. The results showed that IL-2 and IFN-γ were significantly increased in the treatment group compared with the control group. At the same time, IL-4 was decreased, indicating that fusion protein immunization activates T cells and further confirming the tumor suppression effect in vivo (Figure 5F–H).

### 3.6. STEAP1 Knockout Suppresses Immunization Elicited by the Fusion Protein

**Figure 6:**
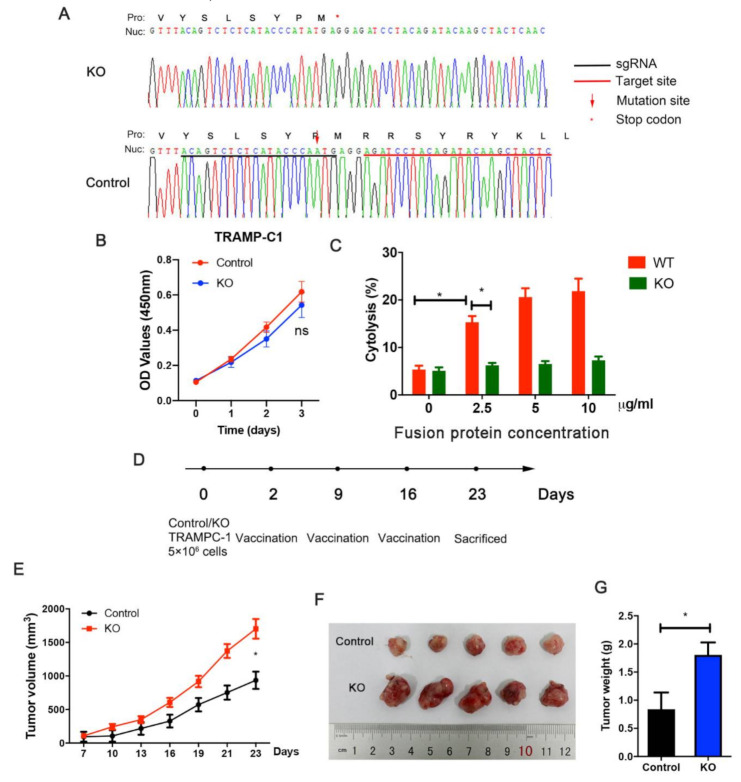
STEAP1 knockout suppresses immunization elicited by the fusion protein. (**A**) TRAMP-C1 cells with STEAP1 knockout (KO) verified using Sanger sequencing. (**B**) The CCK8 assay of wild-type (control) TRAMP-C1 cells and STEAP1-KO TRAMP-C1 cells. (**C**) The LDH assay performed to measure the cytotoxicity of effector T cells against KO cells and control TRAMP-C1 cells at different fusion protein concentrations. (**D**) Schematic diagram for xenograft. (**E**–**G**) Analysis of tumor weight and tumor growth of xenograft. * *p* < 0.05.

To further illustrate the immunization against STEAP1 elicited by the fusion protein vaccine, the epitope was mutated using CRISPR-Cas9. A stop codon was generated before the epitope because of the adenine insertion. The positive cells were screened by sorting monoclonal cells and verified using Sanger sequencing (Figure 6A). The knockout of STEAP1 in TRAMPC-1 cells had a slight effect on cell proliferation but with no statistically significant difference (Figure 6B). An LDH assay in vitro was conducted for the STEAP1(KO) cells and control TRAMP-C1 cells. It was found that the fusion protein could elicit efficient cytotoxicity against control cells but not in KO cells (Figure 6C). The TRAMP-C1 xenograft tumor model with the KO cells and control cells showed that when STEAP-1 is knocked out, the immune protection against the tumor was suppressed (Figure 6D–G).

## 4. Discussion

Two delivery systems were evaluated in a previous study, delivery by STEAP1 cDNA and delivery by virus-like replicon particles (VRPs), which only caused moderately specific CD8 T-cell responses [15]. In this research, we used an Ag85B fusion protein delivery platform for the STEAP1 peptide to enhance the immune response. The Ag85B protein is expressed in all pathogenic mycobacteria and is one of the essential *Mycobacterium tuberculosis* proteins inducing the body’s immune response. Kelly A. et al. showed that BCG deficient in Ag85B showed lower uptake by macrophages, and Ag85B-overexpressing BCG had greater uptake [16]. Ag85B binding to fibronectin (FN) improves immune protection by enhancing uptake by antigen-presenting cells [17,18,19]. FN-binding proteins help the phagocytosis of proteins into antigen-presenting cells (APCs), especially macrophages, monocytes, and DCs [20]. Ag85B also stimulates the production of cytokines, such as TNF-α, and induces strong T cell responses, including cell proliferation and IFN-γ production [21,22,23,24]. Shiki Takamura et al. showed that tumor cells transfected with Ag85B enhance the immunogenicity of tumor-associated antigens [25]. Stimulation with purified Ag85B protein with CFA induces a robust Th1 immune response [26,27,28].

The STEAP1 protein is overexpressed in prostate, bladder, renal, breast, testicular, pancreatic, cervical, and ovarian cancer, making STEAP1 an attractive candidate for a wide range of cancer immunotherapies [15,29,30,31,32,33]. Previous studies have identified a H-2K^b^ epitope STEAP1_186-193_ (RSYRYKLL) [15,34,35,36,37]. A simplex STEAP1 antigen with low immunogenicity has been used in previous studies and only caused a moderate immune response. In this research, we constructed and expressed the fusion protein Ag85B-3×STEAP1_186-193_. Ag85B can present the antigen, enhancing antigen uptake by APCs, thereby inducing effective CTL responses via activating Th1 immunity [12]. The flow cytometry results show that the fusion protein increased the expression of CD11c, CD80, CD86, and MHC II. Furthermore, the cytotoxicity detection assay indicated that the fusion protein elicited CTL responses against STEAP1-expressing cells. We used TRAMP-C1 and RM1 subcutaneous xenograft models to examine the antitumor effects of the fusion protein. The TRAMP-C1 group received four immunizations after tumor inoculation because the TRAMP-C1 tumors grew more slowly, while the RM1 group received three immunizations. The changes in tumor growth and the tumor microenvironment further proved the antitumor effect of the fusion protein. The IFN-γ and IL-2 in the treated groups were significantly higher than those in the control groups, while IL-4 was reduced. High levels of IFN-γ and IL-2 indicate that the fusion protein activated Th1 cells, playing an essential role in antitumor immunity. In addition, IFN-γ upregulates MHC I antigen presentation in tumor cells [38,39,40,41,42]. A low level of IL-4 indicates that Th2 cells were inhibited, which exert immunosuppressive effects [43,44,45]. We created a STEAP1-knockout antigen epitope in TRAMP-C1 cells using CRISPR-Cas9 to illustrate the immune response elicited by the fusion protein. LDH and xenograft tumor assays showed that STEAP1 KO suppresses the CTL response against TRAMP-C1 cells.

In this study, we constructed the fusion protein vaccine using Ag85B and STEAP1_186-193_. However, due to the restriction of the epitope and MHC, the fusion protein vaccine can only induce immune protection against prostate cancer in the C57BL/6 (H-2 b) mouse, which limits the wideness of its applicability. In order to solve this issue, we will choose long antigen peptides to construct a next-generation tumor vaccine. The limitations of the in vivo experiments are that we implanted the prostate cancer cells subcutaneously, not orthotopically, which would have better mimicked the physiological conditions of prostate cancer.

## 5. Conclusions

In summary, our results firstly demonstrate that a fusion protein vaccine with CFA based on the *M. tuberculosis* protein Ag85B and 3×STEAP1_186-193_ induced a CTL response against prostate cancer cells and inhibited tumor growth in vivo. This could be an alternative antigen carrier for inducing antitumor immunotherapy for prostate cancer.

## Figures and Tables

**Table 1:** Primers.

Gene Name	Primer Name	Primer Sequences
STEAP1	F1	AGTCGCTGCCATCATATCATCC
	R1	AAGTCGGAGGCATGTACCATAC
Ag85B + link	F2	CGGCAGCCATATGGCTAGCATGACAGACGTGAGCCGAAAGA
	R2	GTAGGAACGTGATCCTCCACCTCCACCGC
	R3	GGTGGTGGTGGTGCTCGAGCTACAGCAGTTTGTAACGGTAGGAACGC
GAPDH	F4	CATCACTGCCACCCAGAAGACTG
	R4	ATGCCAGTGAGCTTCCCGTTCAG

## Data Availability

Not applicable.

## Supplementary Materials

The following are available online at https://www.mdpi.com/article/10.3390/vaccines9070786/s1, Figure S1: Human STEAP1 expression level in The Human Protein Atlas database. Figure S2: Whole and uncropped western blot.

## Abbreviations

STEAP1	six-transmembrane epithelial antigen of the prostate 1
CTL	cytotoxic T lymphocyte
CRPC	castration-resistant prostate cancer
TAA	tumor-associated antigen
PIN	prostatic intraepithelial neoplasia
LDH	lactate dehydrogenase-releasing cytotoxicity
CFSE	5(6)-carboxyfluorescein diacetate succinimidyl ester
IPTG	isopropyl β-D-1-thiogalactopyranoside
PCR	polymerase chain reaction
CFA	complete Freund’s adjuvant
