# Supplementary material for: Fusion Protein Vaccine Based on Ag85B and STEAP1 Induces a Protective Immune Response against Prostate Cancer
Source: Vaccines (Basel). 2021 Jul 13;9(7):786. doi: 10.3390/vaccines9070786 (PMC8310044; doi:10.3390/vaccines9070786)
Supplement: Supplementary file 1 [file vaccines-09-00786-s001.zip › vaccines-1256631-supplementary.pdf]

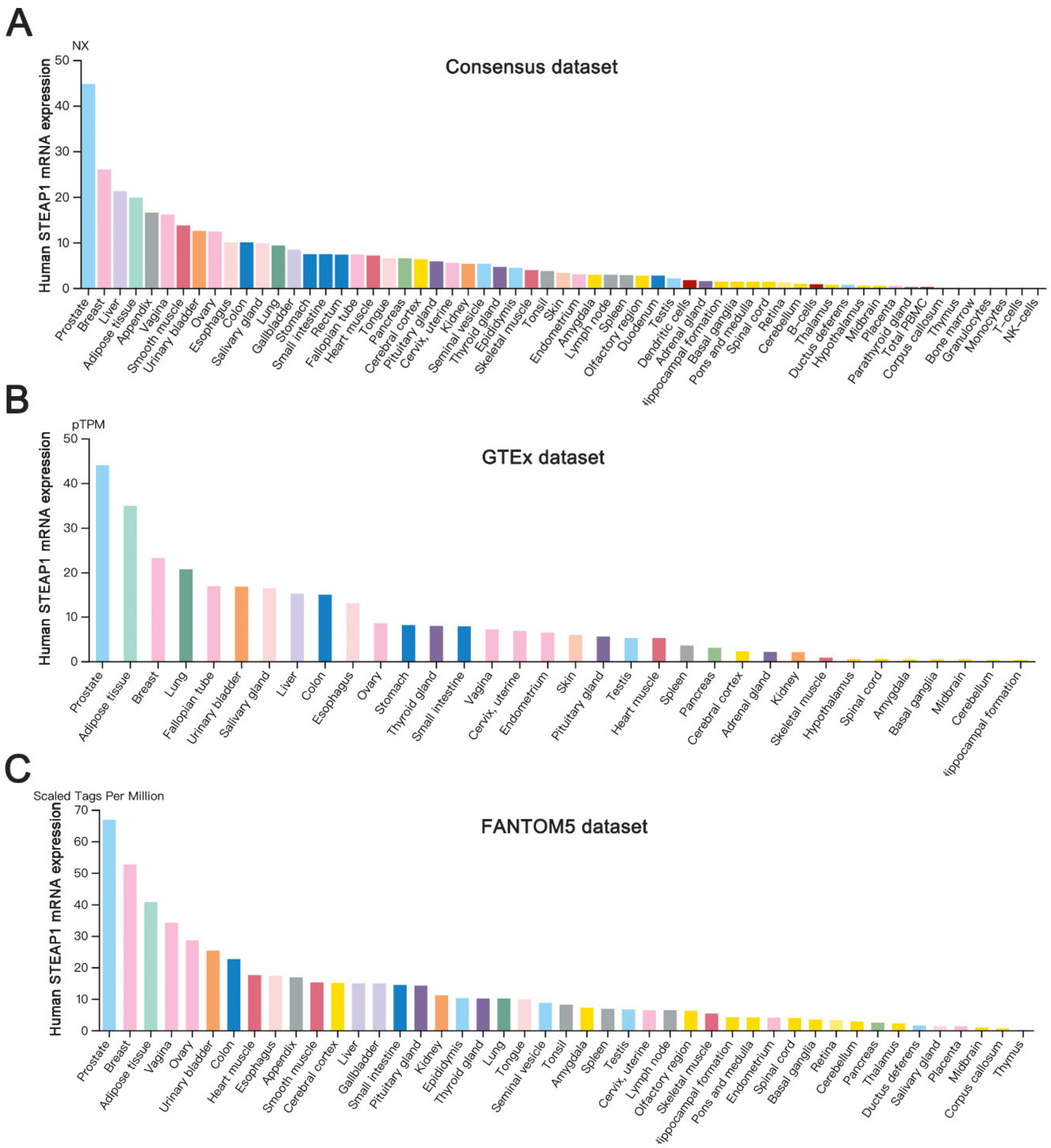

Figure S1. Human STEAP1 expression level in The Human Protein Atlas database. (**A**) Human STEAP1 expression level in consensus dataset of The Human Protein Atlas database. (**B**) Human STEAP1 expression level in GTEx dataset of The Human Protein Atlas database. (**C**) Human STEAP1 expression level in FANTOM5 dataset of The Human Protein Atlas database.

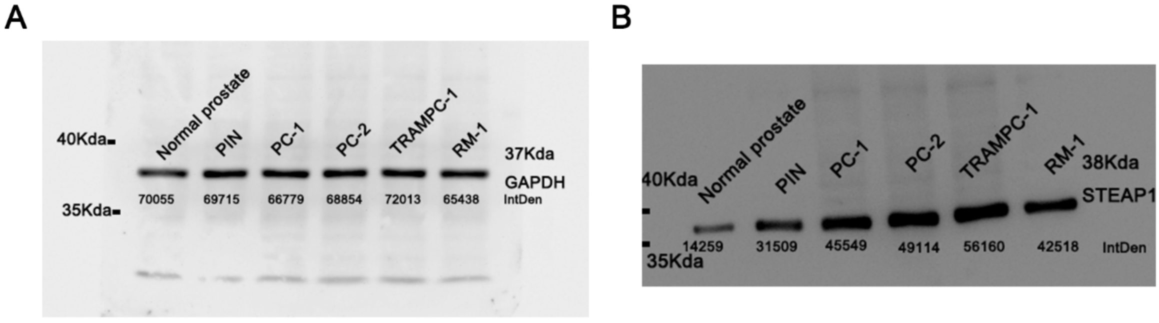

Figure S2. Whole and uncropped western blots. (**A**) western blot of GAPDH for Figure 1C. (**B**) western blot of STEAP1 for Figure 1C. IntDen: Integrated density.
